# The Usefulness of Concomitant Ultrasound Guidance With Surgery for Acute Achilles Tendon Rupture Using an Internal Brace

**DOI:** 10.7759/cureus.79340

**Published:** 2025-02-20

**Authors:** Shuichi Chida, Moto Kobayashi, Tsutomu Sakuraba, Ken Sasaki, Naohisa Miyakoshi

**Affiliations:** 1 Orthopedic Surgery, Hiraka General Hospital, Akita, JPN; 2 Orthopedic Surgery, Hiraka General Hospital, Aktia, JPN; 3 Orthopedic Surgery, Akita University Graduate School of Medicine, Akita, JPN

**Keywords:** acute achilles tendon rupture, early rehabilitation, internal brace, pars, ultrasonography

## Abstract

Background

This study investigated the usefulness of intraoperative ultrasonography in the treatment of acute Achilles tendon rupture (ATR) using an internal brace (Achilles Midsubstance SpeedBridge, Arthrex Inc., Naples, FL), a technique that provides strong internal fixation.

Methodology

Forty-three patients were included and divided into two groups: Group A (*n* = 22), which received ultrasonography, and Group B (*n* = 21), which did not. In Group A, ultrasonography was used during suturing with a specialized jig to confirm the suture needle's position at the center of the proximal stump. Postoperative care in both groups involved initiating active dorsiflexion exercises on the day following surgery and permitting weight-bearing without orthosis once 0° dorsiflexion was achieved. The operative time, Japanese Society for Surgery of the Foot (JSSF) ankle/hindfoot scale, T2-weighted magnetic resonance imaging (MRI) findings at three months postoperatively, and complications were evaluated.

Results

Group A had a significantly shorter operative time (41.9 ± 7.5 minutes vs. 52.1 ± 6.5 minutes, *P *< 0.001) and a lower percentage of high-intensity areas on T2-weighted MRI (1.76% ± 2.68% vs. 8.74% ± 7.02%, *P *< 0.001) compared to Group B. No significant difference was observed in JSSF scale scores (*P *= 0.948). Additionally, no cases of re-rupture or wound infection were reported in either group.

Conclusions

Intraoperative ultrasonography in conjunction with this method may enable precise and reliable suturing, facilitating strong internal fixation and potentially enhancing clinical outcomes.

## Introduction

The treatment of acute Achilles tendon rupture (ATR) has been extensively studied, with several reports detailing conservative and surgical approaches, each yielding varied outcomes [1-4]. While nonoperative management has demonstrated favorable results in many studies [5-7], its high re-rupture rate and delayed recovery have led many surgeons to favor surgical repair [7]. The open technique was considered the gold standard for Achilles tendon repair for decades. However, due to the relatively high incidence of wound complications and adhesions associated with open surgery, percutaneous and minimally invasive techniques have been introduced as alternative approaches to mitigate these issues [8].

In this study, we employed the internal brace technique (Achilles Midsubstance SpeedBridge, Arthrex Inc., Naples, FL), which provides robust internal fixation through a minimally invasive approach [9]. This method involves percutaneous suturing of the proximal stump while leaving the distal stump unsutured. The sutures attached to the proximal stump are directly anchored to the calcaneus using an anchor system. Reliable suturing of the proximal stump is critical, and this can be achieved through a small incision using a Percutaneous Achilles Repair System (PARS) jig [10,11].

Advancements in ultrasonic diagnostic equipment have significantly expanded the use of ultrasonography in the field of musculoskeletal disorders. Ultrasonography has proven to be a valuable tool for diagnosis, treatment, and assessment of therapeutic outcomes [12-14]. In this study, we utilized intraoperative ultrasonographic guidance to ensure precise suturing of the proximal stump during surgery and investigated its clinical utility.

## Materials and methods

This study included 43 patients (43 feet) with a mean age of 44.7 years (range 17-82), who were followed for an average of 18.2 months (range 12-24). Patients were randomly assigned to two groups: Group A (*n* = 22), where surgery was performed with ultrasonographic guidance for suturing the proximal stump, and Group B (*n* = 21), where surgery was performed without ultrasonographic guidance.

**Figure 1:**
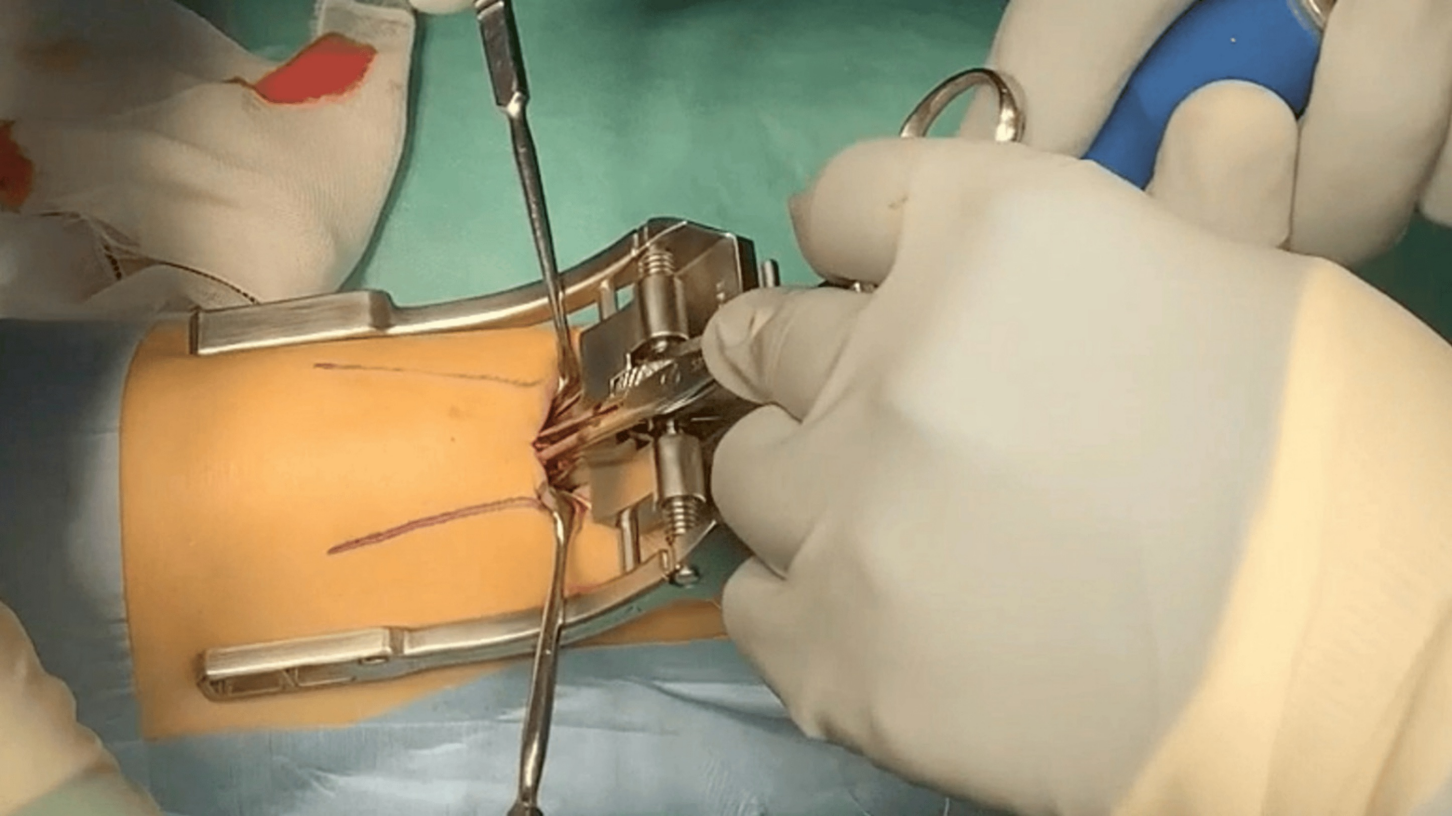
Intraoperative photograph: The Percutaneous Achilles Repair System (PARS) jig was then inserted.

**Figure 2:**
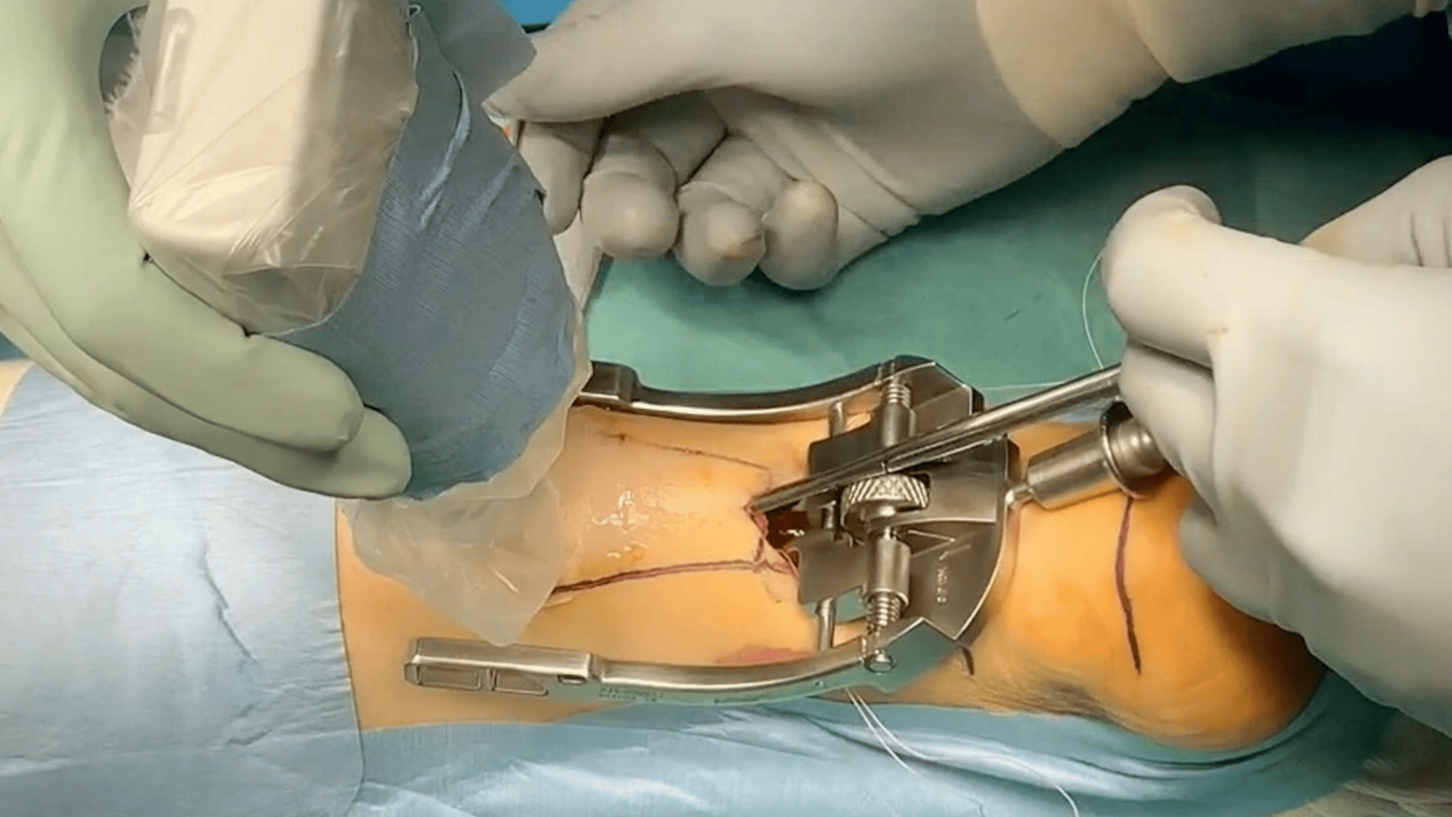
Intraoperative photograph: The needle was passed through the tendon using ultrasonographic guidance.

**Figure 3:**
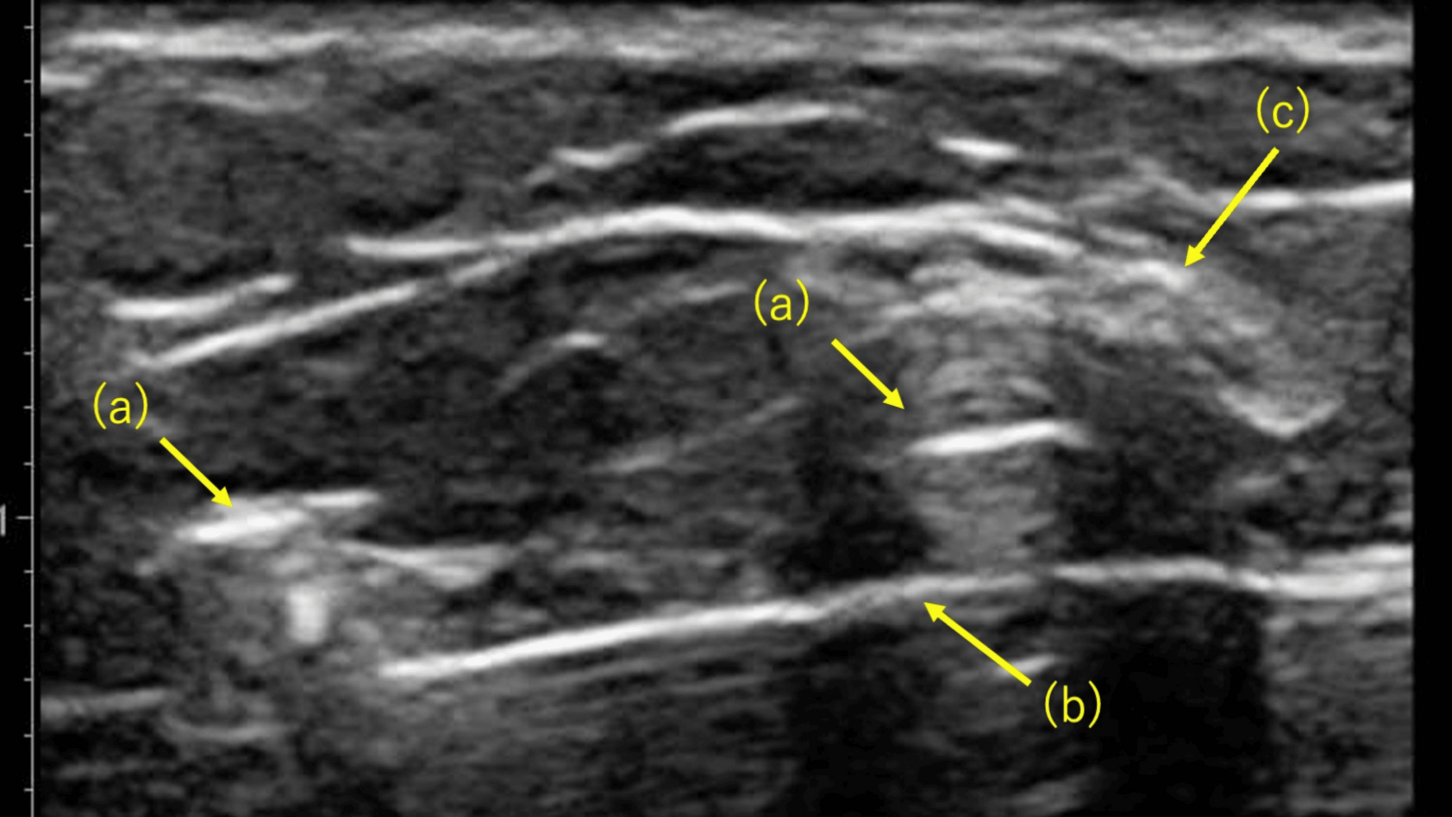
Short-axis view on ultrasonography: (a) The medial arm of the Percutaneous Achilles Repair System (PARS), (b) the needle, and (c) the sural nerve.

**Figure 4:**
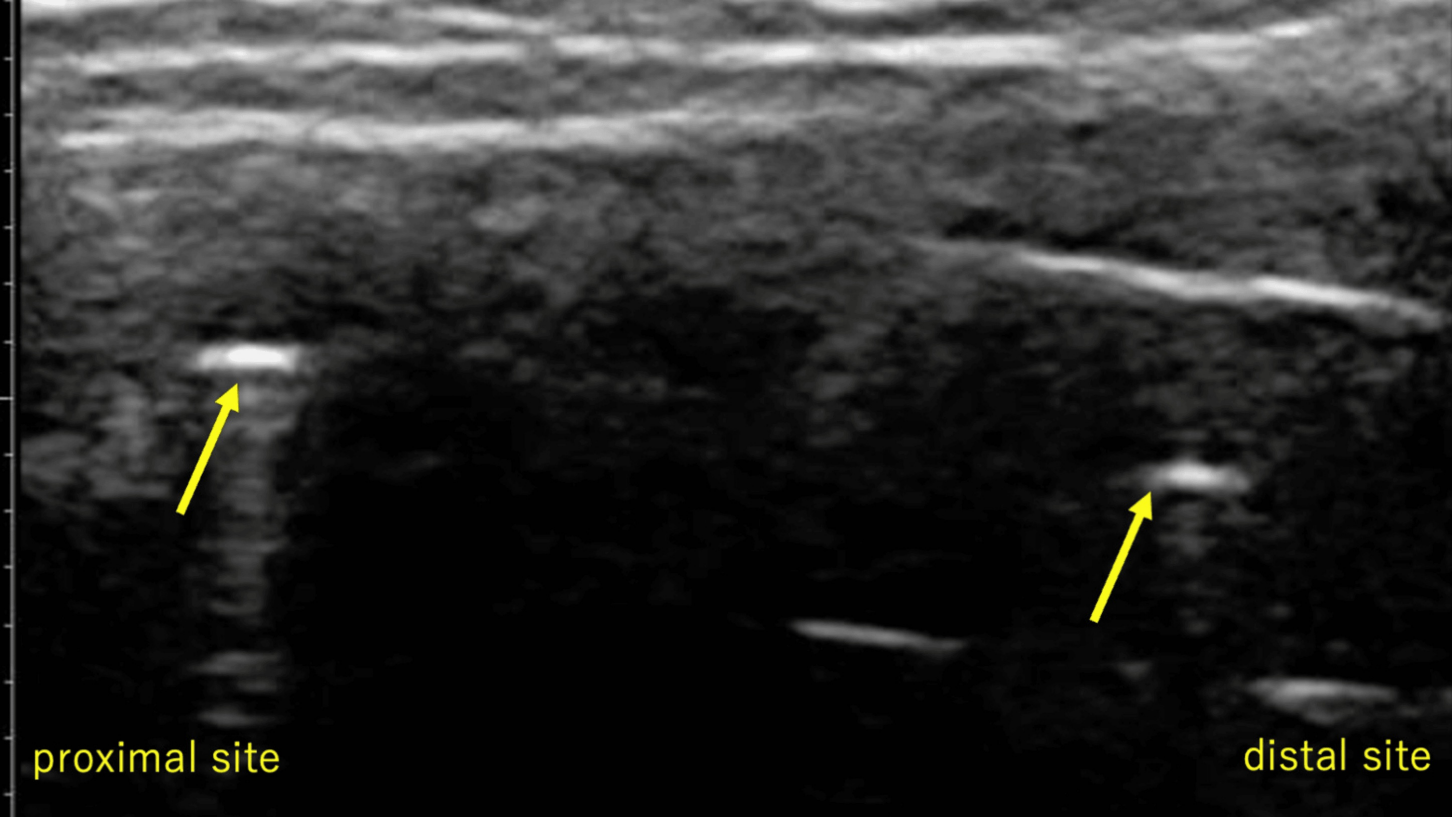
Long-axis view on ultrasonography: Proximal and distal suture needles were present in the center of the Achilles tendon (arrow).

**Figure 5:**
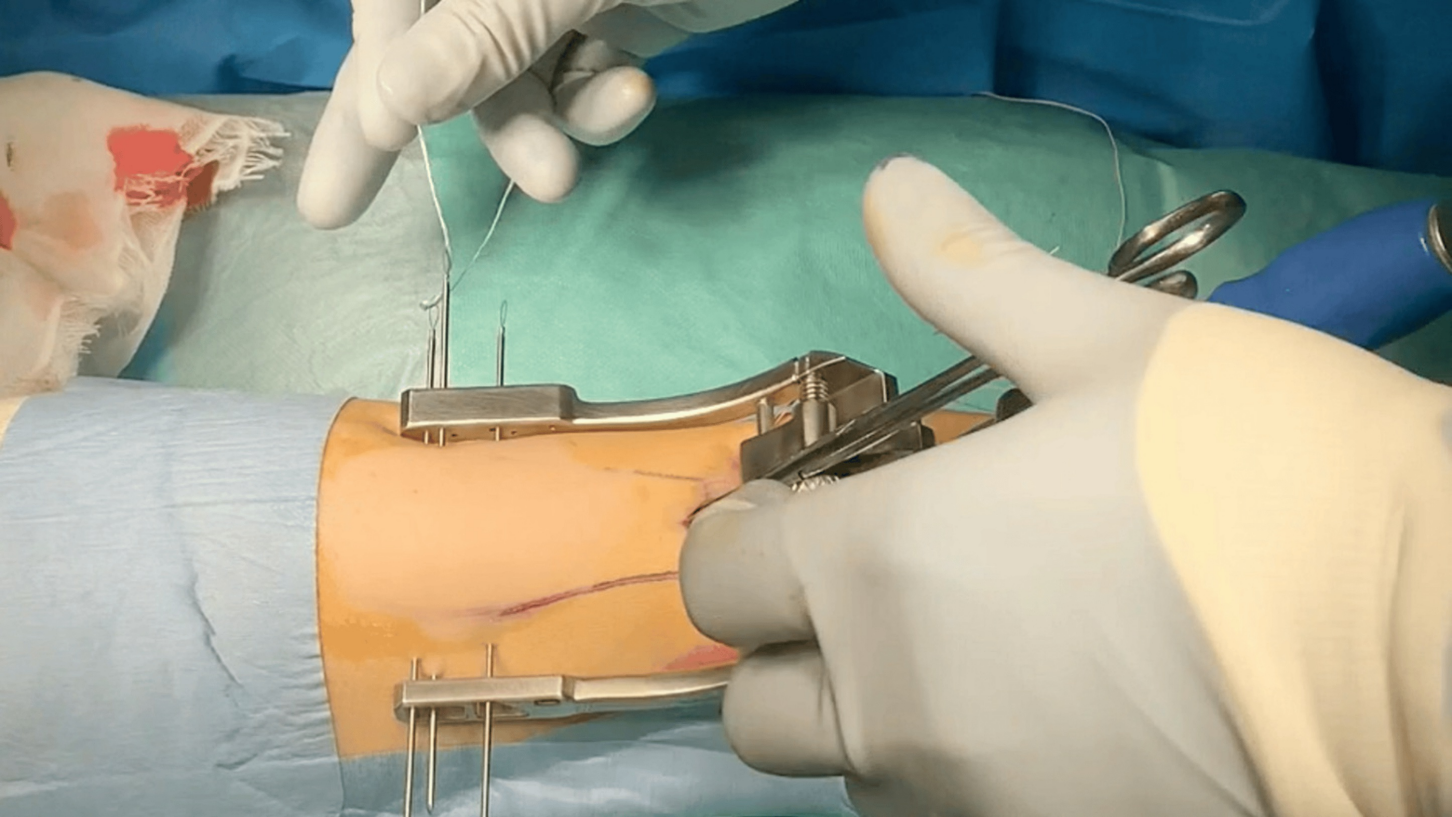
Intraoperative photograph: Additional suture threads were inserted.

Surgery was performed with the patient in the prone position under conduction anesthesia in both groups. A tourniquet was applied to the lower leg. After identifying the proximal stump of the Achilles tendon, a 3-cm longitudinal skin incision was made, centered on the proximal stump. The crural fascia was incised longitudinally, the proximal portion of the tendon was grasped with tendon forceps, and an elevator was used to separate the space between the paratenon and the tendon. The medial arm of the PARS jig system was then inserted into the paratenon (Figure 1). Specialized needles were used to pass five suture threads through the tendon. In Group A, the most proximal and distal suture needle positions were confirmed to be in the center of the stump using ultrasonographic guidance (Figures 2-4). Subsequently, additional suture threads were sequentially inserted between the proximal and distal sutures (Figure 5). Ultrasonographic devices, including the ACUSON Freestyle (Siemens, Munich, Germany) and Venue 50 (GE Healthcare UK Ltd., Buckinghamshire, UK), were utilized for this purpose. The suture threads were passed conventionally, with two non-locking sutures and one locking suture applied.

**Figure 6:**
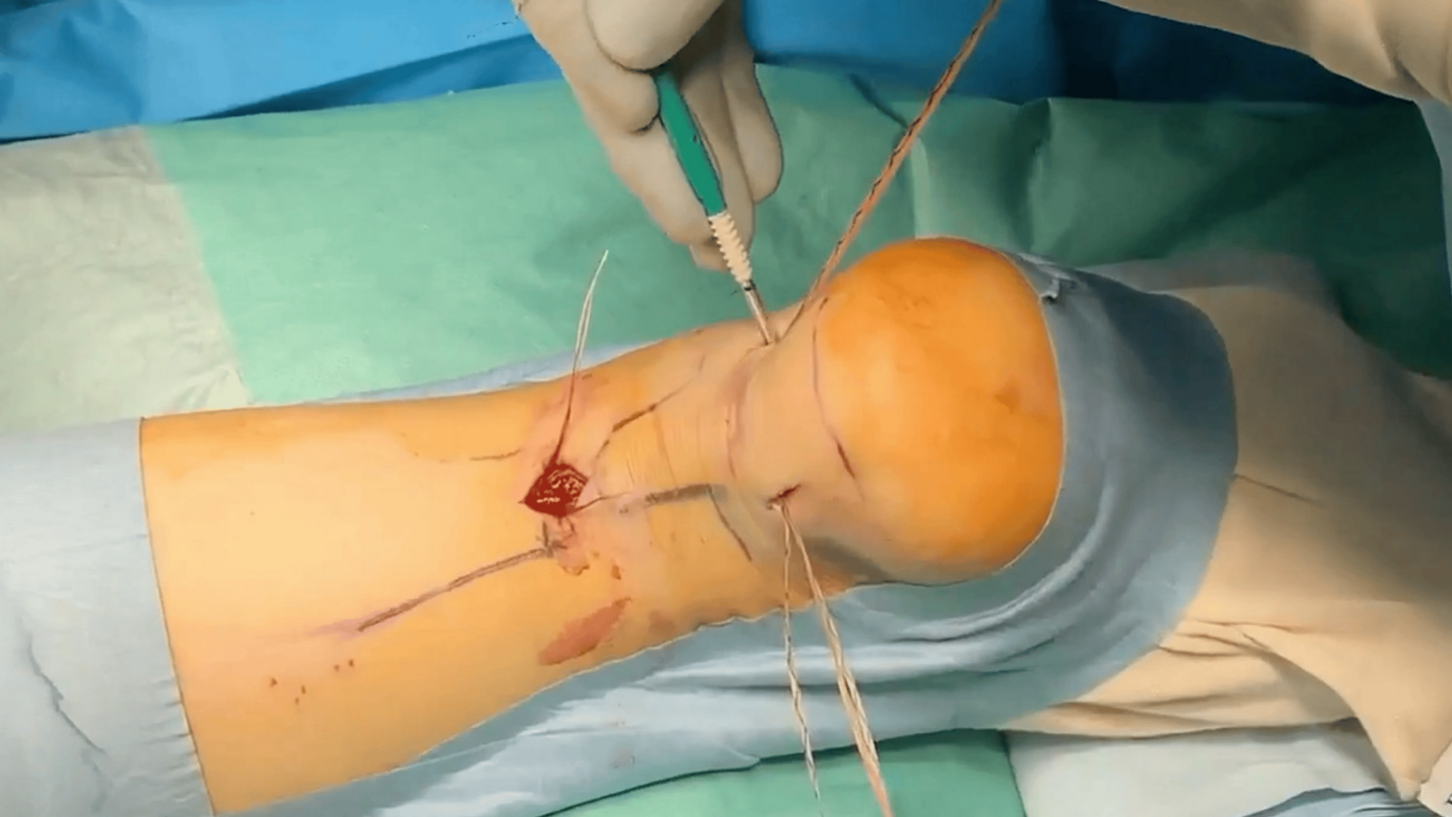
Intraoperative photograph: Three suture threads were guided and anchored to the calcaneus using polyether ether ketone (PEEK) SwiveLock® anchors (Arthrex Inc., Naples, FL).

Next, two additional skin incisions were made posterior-medially and posterior-laterally, distal to the Achilles tendon insertion site. Three suture threads were guided and anchored to the calcaneus using PEEK SwiveLock® anchors (Arthrex Inc.) (Figure 6). During fixation, the stumps were pulled together under direct visualization to ensure proper contact and were fixed with the ankle positioned at 30° of plantar flexion. No stump suturing or paratenon repair was performed; however, the crural fascia was securely repaired.

Postoperative management was identical in both groups. On the day after surgery, the cast was modified to a plaster shell, and active dorsiflexion exercises were initiated. Weight-bearing walking was permitted once 0° dorsiflexion was achieved. No orthoses were used postoperatively. Jogging was allowed at six to eight weeks, with a target of full return to sports by three months postoperatively.

**Figure 7:**
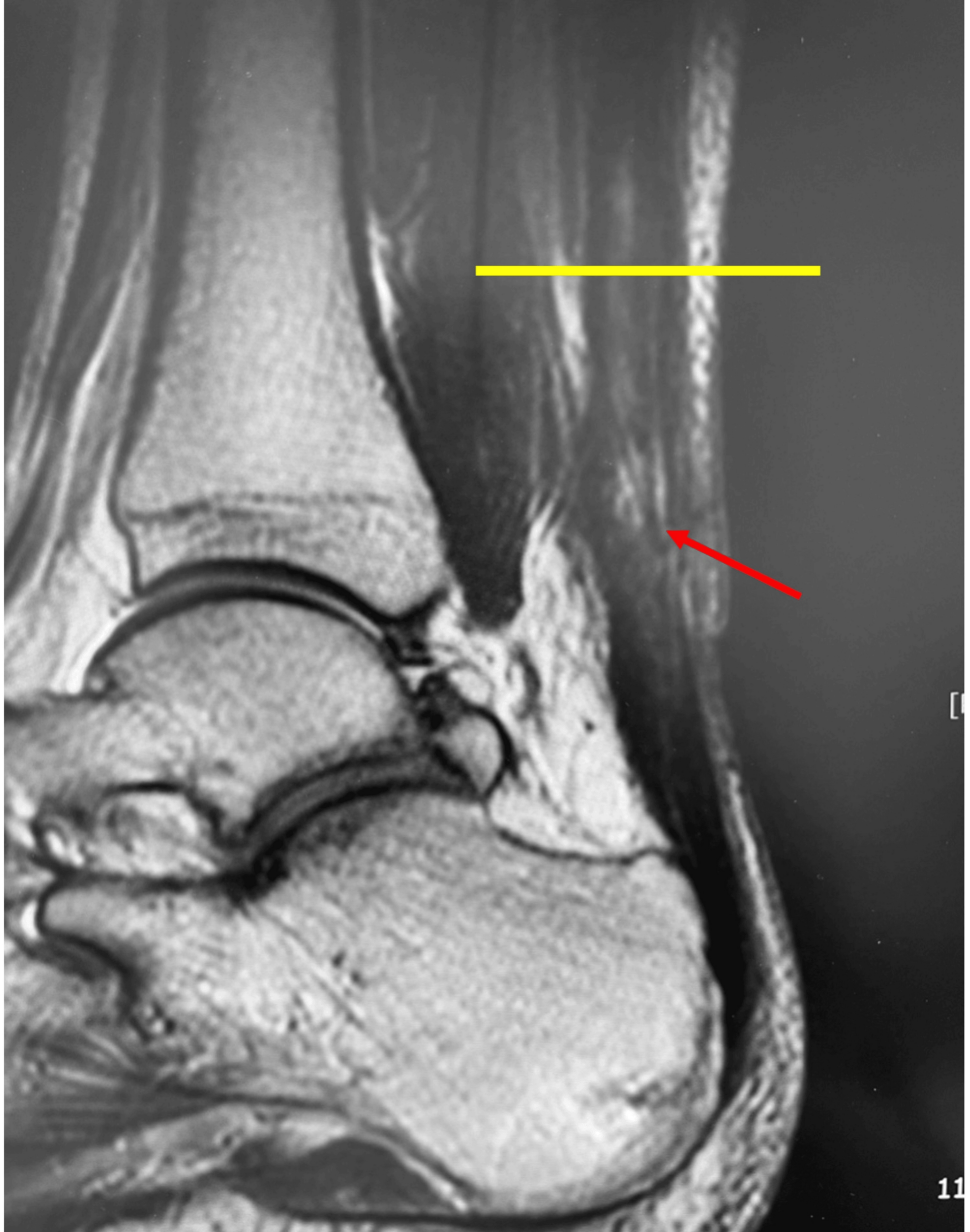
Sagittal view on T2-weighted MRI. The rate of the high-intensity area (red arrow) was calculated by dividing the area located at the musculotendinous junction (yellow line) over the distal Achilles tendon by the entire area.

The evaluation parameters included operative time, the Japanese Society for Surgery of the Foot (JSSF) ankle/hindfoot scale scores at the final follow-up [15,16], the percentage of high-intensity areas in the Achilles tendon on T2-weighted MRI at three months postoperatively, and complications. The percentage of high-intensity areas was calculated by dividing the high-intensity area by the total area of the Achilles tendon from the musculotendinous junction to the calcaneal insertion site in a sagittal T2-weighted MRI slice centered on the Achilles tendon (Figure 7). Area measurements were performed using the NEOVISTA I-PACS CX measurement tool (Konica Minolta Inc., Tokyo, Japan). Each measurement was repeated twice by the same examiner, and the average value was recorded.

All statistical analyses were performed using EZR (Jichi Medical University, Tochigi, Japan), a modified version of R Commander with enhanced biostatistical functions [17]. Continuous variables were compared using the independent t-test, while categorical variables were analyzed using either Fisher’s exact test or the chi-square test, depending on sample size constraints. A *P*-value < 0.05 was considered statistically significant.

## Results

The mean age was 52.7 ± 17.6 years in Group A and 37.2 ± 10.0 years in Group B, showing a statistically significant difference (*t *= 3.423, *P *< 0.01). The mean body mass index (BMI) was 24.2 ± 4.9 kg/m² in Group A and 26.6 ± 3.3 kg/m² in Group B, also demonstrating a significant difference (*t *= -2.577, *P *< 0.01). No significant difference was observed in the male-to-female ratio (Group A = 18:4; Group B = 16:5), as determined by Fisher’s exact test (*P *= 0.398) (Table 1).

**Table 1:** Comparison of demographic data between Groups A and B. Age, body mass index (BMI), and follow-up duration were analyzed using an independent t-test, while sex distribution was analyzed using a chi-square test. Values are expressed as mean ± standard deviation. 95% CI, 95% confidence interval; OR, odds ratio

	Group A	Group B	Statistics	95% CI	*P*-value
Patients (*n*)	22	21	-		-
Age (years)	52.7 ± 17.6	37.2 ± 10.0	*t *= 3.423	6.15-23.92	<0.01
Sex (male:female)	18:4	16:5	*χ*² = 0.023	OR = 0.67; 95% CI = 0.11-3.76	0.878
Body mass index (kg/m^2^)	24.2 ± 4.9	26.6 ± 3.3	*t *= -2.577	-4.32 to -0.52	<0.01
Follow-up (months)	14.0 ± 5.5	13.8 ± 12.6	*t *= 0.085	-5.62 to 6.12	0.850

The operative time was significantly shorter in Group A (41.9 ± 7.5 minutes) compared to Group B (52.1 ± 6.5 minutes, *t *= -4.623, *P* < 0.001). On T2-weighted MRI three months postoperatively, the percentage of high-intensity areas in the Achilles tendon was 1.76% ± 2.68% in Group A and 8.74% ± 7.02% in Group B, indicating a significantly lower intensity in Group A (*t *= -4.035, *P *< 0.001). There was no significant difference in JSSF scale scores between the two groups (*t *= -0.383, *P *= 0.948). Additionally, no cases of re-rupture, wound infection, or sural nerve injury were reported in either group. However, two cases of anchor removal were observed in Group B, whereas none occurred in Group A (Fisher’s exact test, *P *= 0.222) (Table 2).

**Table 2:** Comparison of intraoperative and postoperative outcomes between Group A and Group B. Operative time, T2 high intensity, and the Japanese Society for Surgery of the Foot (JSSF) scale were analyzed using an independent t-test. The removal of the anchor rate was analyzed using Fisher’s exact test. Since no cases of anchor removal were observed in Group A, the odds ratio (OR) was calculated as infinite (Inf), and the 95% confidence interval (CI) was not estimable. Values are expressed as mean ± standard deviation.

	Group A		Group B	Statistics	95% CI	*P*-value
Operative time (minutes)	41.9 ± 7.5	52.1 ± 6.5		*t *= -4.623	-15.63 to -6.13	<0.001
T2 high intensity (%)	1.76 ± 2.68	8.74 ± 7.02		*t *= -4.035	-10.49 to -3.46	<0.001
JSSF scale (points)	97.8 ± 4.4	97.9 ± 3.7		*t *= -0.383	-2.99 to 2.03	0.948
Complications						
Re-rupture	0	0		-		-
Wound infection	0	0		-		-
Sural nerve injury	0	0		-		-
Removal of anchor	0	2		Fisher’s exact test, OR = Inf	95% CI = 0.20 to Inf	0.222

## Discussion

Hsu et al. reported that ATRs treated with PARS had comparable overall outcomes to those treated with open repair [10]. They found that a greater proportion of patients treated with PARS were able to return to baseline physical activities within five months compared to those who underwent open repair. Additionally, there were no significant differences between the groups in rates of re-rupture, sural neuritis, wound dehiscence, superficial and/or deep infection, or reoperation. Macaluso et al. conducted a cadaveric and finite element (FE) model biomechanical study to compare the mechanical differences between PARS and Krackow open repair under tensile load and rotation [18]. Their findings demonstrated that, based on biomechanical parameters observed through mechanical testing and FE analysis, PARS was superior to the Krackow repair in terms of greater strength, higher failure force, and lower gap formation. Therefore, limited-incision techniques for acute ATRs have been developed in recent years to enhance recovery and reduce postoperative complications compared with traditional open repair.

Treatment with an internal brace is a surgical procedure in which the proximal stump is percutaneously sutured, with the sutures directly anchored to the calcaneus. This technique differs from conventional invasive suture methods. McWilliam and Mackay reported that this method provides stronger internal fixation compared to the traditional Krackow suture, with a mean time to return to pre-injury activity levels of 18.2 weeks [9]. Furthermore, we have previously demonstrated that surgical treatment of ATR using an internal brace enables early rehabilitation without the need for orthosis, facilitates a safe and early return to daily activities, and minimizes complications, including re-rupture.

One significant advantage of this method is that the use of a specialized jig allows suturing of the proximal stump through a small incision, enabling strong fixation without directly suturing the stump [9]. Since the stump is not sutured, thickening at the sutured region is avoided, thereby reducing the risk of infection associated with invasive sutures. In this study, no infections were reported among the patients.

However, ensuring reliable suturing of the proximal stump is critical, as it is sutured percutaneously while the distal threads are securely anchored to the calcaneus. Several studies have highlighted the usefulness of intraoperative ultrasonography in the percutaneous repair of ATR [19-21]. Therefore, we utilized intraoperative ultrasonographic guidance to ensure precise suturing of the proximal stump during surgery. Although no re-ruptures occurred in either group and the clinical outcomes were comparable, the rate of high-intensity areas in the Achilles tendon on T2-weighted MRI at three months was significantly lower in the ultrasonography-guided group (2%) compared to the non-ultrasonography group. This finding suggests that ultrasonography-guided suturing of the proximal stump contributes to reliable internal fixation, thereby enhancing the repair and union of the ruptured region.

Yongliang et al. reported that minimally invasive repair assisted with intraoperative ultrasonography can reduce surgical time and complications, particularly sural nerve injury [20]. Similarly, in this study, the operative time was significantly shorter in the ultrasonography-guided group. Real-time confirmation of the suture thread insertion position likely prevented misplacement and reduced the need for re-insertion. While percutaneous suturing raises concerns regarding potential sural nerve impairment, no such complications were observed in this study. Notably, the proximity of the suture needle to the sural nerve necessitates caution. Ultrasonography allows real-time visualization of the sural nerve, contributing to the safe execution of this technique.

This study has several limitations. Postoperative evaluations of suture threads and Achilles tendon length were not conducted, and the high-intensity area on T2-weighted MRI was measured using only a single sagittal slice. Further research is warranted to address these limitations.

## Conclusions

Ultrasonography-guided suturing of the proximal stump enables precise needle placement, ensuring reliable internal fixation and improved surgical accuracy. MRI findings demonstrate a significant reduction in high-intensity areas at the ATR site, suggesting its potential to accelerate tendon healing. Additionally, this technique significantly reduces operative time compared to conventional methods, likely due to more precise suture placement and a decreased need for re-insertion.

Furthermore, ultrasonographic guidance enhances procedural safety by allowing real-time visualization of critical anatomical structures, such as the sural nerve, thereby minimizing the risk of nerve injury. Given its advantages in enhancing fixation strength, reducing complications, and facilitating early rehabilitation, intraoperative ultrasonography may serve as a valuable adjunct in ATR repair. Future studies with larger cohorts and long-term follow-up are needed to further validate these findings and optimize surgical protocols.
